# A Red Fluorescent Protein Reporter System Developed for Measuring Gene Expression in Photosynthetic Bacteria under Anaerobic Conditions

**DOI:** 10.3390/microorganisms10020201

**Published:** 2022-01-18

**Authors:** Mingyue Jiang, Yan Zeng, Lingwei Cui, Mengmei Wang, Yanning Zheng

**Affiliations:** 1 State Key Laboratory of Microbial Resources, Institute of Microbiology, Chinese Academy of Sciences, Beijing 100101, China; jiangmingyue16@mails.ucas.ac.cn (M.J.); zengy@im.ac.cn (Y.Z.); cuilingwei21@mails.ucas.ac.cn (L.C.); wangmengmei20@mails.ucas.ac.cn (M.W.); 2 College of Life Sciences, University of Chinese Academy of Sciences, Beijing 100101, China

**Keywords:** red fluorescent protein, anaerobic reporter system, nitrogenase, gene regulation, photosynthetic bacteria, *Rhodopseudomonas palustris*

## Abstract

The photosynthetic bacterium *Rhodopseudomonas palustris* converts nitrogen gas (N_2_) to fertilizer ammonia (NH_3_) and also produces clean energy hydrogen gas (H_2_) from protons (H^+^) when it is grown anaerobically in nitrogen fixing medium with illumination, a condition that promotes the expression of active nitrogenase. Compared with quantitative real-time PCR (qRT-PCR) and the *lacZ* reporter system, two methods commonly used for in vivo study of nitrogenase regulation in photosynthetic bacteria, the fluorescent protein reporter system has advantages in terms of its simplicity and sensitivity. However, little is known concerning if the fluorescent protein reporter system can be used in bacterial cells that need to grow anaerobically. Here, we developed an RFP-based method to measure the nitrogenase gene expression in photosynthetic bacteria grown anaerobically. This method was able to determine the levels of both the genome-based and the plasmid-based nitrogenase expression under anaerobic conditions, providing a better method for in vivo study of gene expression affected by oxygen. The RFP reporter system developed here will promote a better understanding of the molecular mechanism of nitrogenase regulation and will be used on other genes of interest in a wider range of anaerobic bacteria.

## 1. Introduction

*Rhodopseudomonas palustris* is a photosynthetic nitrogen-fixing bacterium widely distributed in nature [1]. Diazotrophs convert atmospheric nitrogen gas (N_2_) to ammonia (NH_3_) by nitrogenase as a major source of bioavailable nitrogen. Moreover, the conversion of N_2_ to NH_3_ by nitrogenase is accompanied by the production of hydrogen gas (H_2_), which can be used as clean energy [2]. Nitrogenase can be further divided into three different categories: molybdenum-iron (MoFe) or Mo nitrogenase, vanadium-iron (VFe) or V nitrogenase, and iron-iron (FeFe) or Fe-only nitrogenase, each differentiated by the metal composition in the active site cofactor [3,4]. In addition to nitrogen fixation and hydrogen production, Fe-only nitrogenase is capable of reducing carbon dioxide (CO_2_) to methane (CH_4_), bringing a much stronger greenhouse gas (CH_4_) to the atmosphere and causing an acceleration of global warming [5]. *R. palustris* is able to express all three nitrogenases, making it an attractive model bacterium for in vivo studies of nitrogenase [5,6]. However, nitrogenase expression is tightly regulated by environmental conditions such as oxygen level, nitrogen availability, and metal content. *R. palustris* expresses nitrogenase and fixes nitrogen only when grown anaerobically with illumination (although it grows well aerobically in the medium supplemented with fixed nitrogen such as ammonia). Therefore, a simple and reliable method that works well under anaerobic conditions could benefit the in vivo studies of nitrogenase regulation in photosynthetic bacteria.

**Figure 1:**
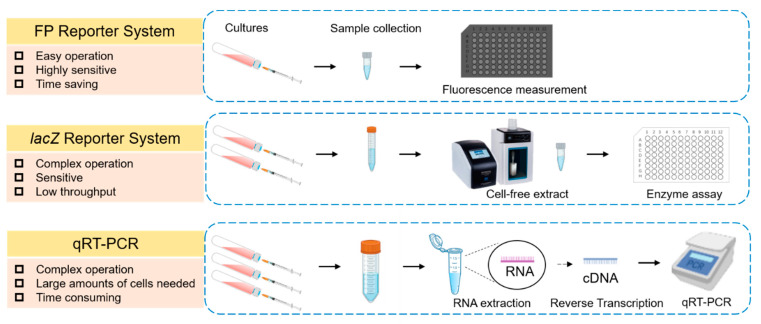
Comparison of fluorescence reporter system, *lacZ* reporter system, and qRT-PCR in terms of measuring gene expression. The advantages and disadvantages of three commonly used methods for measuring levels of gene expression in bacterial cells are summarized. The fluorescent protein (FP) reporter system determines gene expression by detecting fluorescence signals generated by fluorescent proteins. The gene product of *lacZ* is β-galactosidase, which can cleave the glycosidic bond in substrate X-gal and finally produce an intense blue chemical (5,5′-dibromo-4,4′-dichloro-indigo) to quantify target gene expression. The method of qRT-PCR measures the levels of mRNA by firstly converting mRNA into cDNA using a reverse transcriptase and then quantifying cDNA using real-time PCR.

Quantitative real-time PCR (qRT-PCR) is a regular method to measure the expression of target genes [7]. In this method, RNA is firstly extracted from the bacterial cells and then transcribed into complementary DNA (cDNA) by reverse transcriptase. The cDNA is finally used as the template for real-time PCR reaction, with a housekeeping gene employed as an internal reference to control for error between samples (Figure 1). Given that RNA molecules can be easily degraded and the integrity of RNA molecules is quite important for subsequent real-time PCR experiment (which reflects the gene expression levels at the moment of sampling), it requires to take a lot of time and effort to reduce the RNA degradation during the preparation of RNA samples [8]. The *lacZ* gene encoding β-galactosidase is another reporter gene that is commonly used in photosynthetic bacteria [9,10,11]. A colorimetric assay is used to determine the activity of β-galactosidase, which is responsible for the degradation of β-galactosyl linkages [12,13]. Given that a certain number of cells is required to prepare the cell-free extract for the colorimetric assay, this method is unsuitable to measure the gene expression in situations where the cell densities are low [14].

Reporter systems are powerful tools for investigating transcriptional regulation. Green fluorescent protein (GFP), which is the first fluorescent protein being used to measure gene expression in prokaryotes and eukaryotes, was first isolated from *Aequorea victoria* in 1960 [15,16,17,18,19]. After a series of mutant screening and genetic modification [20,21,22], fluorescent proteins such as mCherry, mStrawberry, and tdTomato have been widely used for gene expression measurement, protein localization, in situ screening, and multiomic profiling [23]. As the Discosoma Red fluorescent protein (DsRed) variant mCherry has the best photostability, the fastest maturation, the higher N-terminal fusion tolerance and the excellent pH resistance (Figure 1), it has become the most widely used red fluorescent protein (RFP) [23]. However, a significant limitation of fluorescent proteins is their requirement of molecular oxygen (O_2_) for the maturation of the fluorophore [14]. Little is known to date about if the fluorophore can be fully matured after exposing the cells grown anaerobically to oxygen, and it is still unclear if nitrogenase expression correlates well with the fluorescence intensities recovered from anaerobic conditions.

Here, we describe a simple and highly sensitive method capable of determining the nitrogenase gene expression by RFP reporter system in photosynthetic bacteria grown anaerobically. This method can be used to measure both the genome- and the plasmid-based nitrogenase gene expression and is also able to complete the analysis of large amounts of samples with tiny amounts of biomass in a short time.

## 2. Materials and Methods

### 2.1. Bacterial Strains and Growth Conditions

For genetic manipulations, *R. palustris* strains were grown aerobically on defined mineral medium (PM) agar supplemented with 10 mM succinate at 30 °C [24]. *Escherichia coli* S17-1 was grown in LB medium at 37 °C. When appropriate, *R. palustris* was grown with gentamicin at 100 μg mL^−1^. *E. coli* cultures were supplemented with gentamicin at 20 μg mL^−1^. *R. palustris* strains and *R. rubrum* UR2 were grown anaerobically in nitrogen-fixing medium (NFM) [2]. This defined mineral medium is the same as PM medium but lacks ammonium sulfate. N_2_ gas was provided in the headspace of sealed culture tubes. 20 mM acetate was included as the carbon source and the medium was supplemented with 10 μM sodium molybdate (Na_2_MoO_4_). All cultures except *E. coli* strains were incubated anaerobically with illumination.

### 2.2. Genetic Manipulation of R. palustris and R. rubrum

All strains and plasmids used are listed in Table 1. In-frame insertion of the *mCherry* gene was created by PCR using the Q5 high-fidelity DNA polymerase to amplify three DNA fragments including upstream and downstream of the stop codon for *nifK* and *mCherry* gene with RBS. These fragments were then incorporated into *Pst*I-digested pJQ200SK suicide vector using the T5 exonuclease-dependent assembly system [25]. Plasmid pJQ-nif-RFP was mobilized into *R. palustris* CGA009 and *R. palustris* CGA676 by conjugation with *E. coli* S17-1, and double crossover events for allelic exchange were achieved using a selection and screening strategy as described previously [26]. For the overexpression of RFP, Q5 high-fidelity DNA polymerase was used to amplify *mCherry* gene by PCR. The amplified *mCherry* was then incorporated into the *EcoRI* site of the broad-host-range plasmid pBBR1MCS-5 [27]. After corresponding promoter was cloned into the *SacI* site, the resulting recombinant plasmid was mobilized into *R. palustris* CGA009, *R. palustris* CGA676 or *R. rubrum* UR2 by conjugation with *E. coli* S17-1. All engineered strains were verified by PCR and sequencing.

### 2.3. Measurement of Fluorescence Intensity and Cell Growth

Cultures in log phases were harvested by centrifuging at 12,000 rpm for 1 min (Eppendorf Centrifuge 5424R, Eppendorf AG, Hamburg, Germany). The cell pellets were washed twice with 10 mM phosphate buffer (PBS). The cells were pelleted by centrifuging at 12,000 rpm for 1 min, and then were resuspended with 10 mM PBS. A 200 μL resuspended culture was then transferred to the 96-well clear and black plates for the measurement of OD_660 nm_ and relative fluorescence intensity by a microplate reader (Synergy TMH4, BioTek Instruments, Winooski, VT, USA), respectively. When measuring the relative fluorescence of RFP, the excitation and emission wavelengths were set to 587 nm and 610 nm, respectively.

### 2.4. Quantitative Real-Time PCR Analysis

The mRNA expression levels of *nifD* gene encoding a subunit of molybdenum nitrogenase were measured by qRT-PCR in different growing stages of *R. palustris* strains. *R. palustris* cells were firstly harvested from cultures grown to the early, mid and late log phases, respectively. After RNA extraction, cDNA was then prepared with 2 μg total RNA using FastKing-RT SuperMix (TIANGEN Biotech, Beijing, China) in a 20 μL reaction mixture, which was incubated at 42 °C for 15 min to remove the genomic DNA and complete the reverse transcription. Another incubation at 95 °C for 3 min was carried out to stop the enzyme reaction. Reactions were carried out using SuperReal PreMix Plus (TIANGEN Biotech, Beijing, China) with 50 ng cDNA and 0.5 mM of each primer (Table 1) in a final volume of 20 μL, with cycling parameters (40 cycles of 95 °C for 10 s, 60 °C for 20 s, and 72 °C for 32 s) set per optimized cycling conditions for the LightCycler 480 II Real-Time PCR System (Roche Diagnostics, Basel, Switzerland). Transcriptional levels of the genes tested were normalized to the transcriptional levels of housekeeping gene *rpoD* [7].

### 2.5. Examination of the Spectroscopic Properties of mCherry Fluorescent Protein

*R. palustris* and *E. coli* strains overexpressing mCherry fluorescent protein were grown under aerobic or anaerobic conditions, as indicated in the figure or the text. Cultures in log phase were harvested by centrifuging at 12,000 rpm for 1 min. The cell pellets were washed twice with 10 mM PBS, and then resuspended with the same buffer. A 200 µL cell suspension was transferred to a 96-well black microplate to examine the spectroscopic properties of mCherry fluorescent protein. When the wavelength of excitation monochromator was set to 587 nm, the emission spectrum ranging from 510 nm to 710 nm was recorded. Inversely, the excitation spectrum ranging from 447 nm to 647 nm was recorded when the wavelength of emission monochromator was set to 610 nm.

## 3. Results

### 3.1. Influence of Oxygen Exposure Time on Fluorescence Intensity of RFP in R. palustris

**Figure 2:**
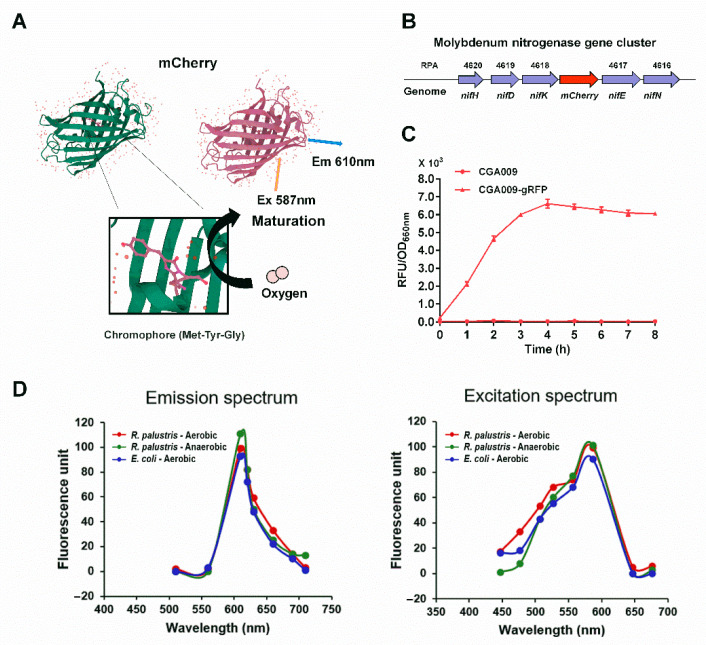
Fluorescence of RFP could gradually recover after *R. palustris* cells grown anaerobically were exposed to air. Molecular oxygen is required for the maturation of the mCherry chromophore (Met-Tyr-Gly), which limits its application in anaerobic bacteria (**A**). To examine whether mCherry can be used as a fluorescence reporter to measure the expression levels of Mo nitrogenase, which is encoded by the *nifHDK* genes, a fluorescence reporter strain CGA009-gRFP (CGA3005) was constructed by inserting the *mCherry* gene into the downstream of *nifK* gene of *R. palustris* genome (**B**). The fluorescence intensities of RFP reached the maximum after the bacterial cells were taken out of the anaerobic tubes for about four hours, and the maximal fluorescence intensity can be maintained for a couple of hours. These data are the average of three independent experiments, and the error bars represent the standard deviation (**C**). No differences in emission and excitation spectra were observed among bacterial cells (*R. palustris* and *E. coli*) grown aerobically and anaerobically when corresponding wavelengths of excitation and emission monochromators were set to 587 nm and 610 nm, respectively (**D**).

*R. palustris*, one of the most important model organisms for in vivo studies of nitrogenases, generates a large amount of ATP required for nitrogen fixation from light energy under anaerobic conditions [1,32,33]. To make RFP capable of detecting the expression of nitrogenase as a reporter system, we inserted the *mCherry* gene with ribosome binding site (RBS) into the genome of *R. palustris* CGA009 immediately downstream of the *nifHDK* gene cluster encoding Mo nitrogenase. The generated *nifHDK-mCherry* gene cluster is transcribed into a single polycistronic mRNA from the *nifH* promoter where initiation of transcription is tightly regulated (Figure 2B). The mutant strain CGA3005 expressing RFP was incubated anaerobically with illumination. However, the maturation of RFP requires the participation of O_2_ (Figure 2A). To examine if RFP still can fluoresce when it is switched from anaerobic to aerobic environments, *R. palustris* CGA3005 cells expressing RFP were taken from the sealed culture tubes and their fluorescence intensities were measured. The fluorescence intensities of *R. palustris* CGA3005 gradually increased with oxygen exposure time, and reached the maximum after a 4-h oxygen exposure at room temperature. More importantly, the maximal fluorescence intensity can stably maintain for at least four hours, providing much greater convenience for measuring the fluorescence intensities of RFP (Figure 2C). To further examine if the mCherry fluorescent protein can authentically fluoresce when it is switched from anaerobic to aerobic conditions, we compared the spectroscopic properties of *R. palustris* and *E. coli* cells that overexpress mCherry with a strong promoter (J23119) under aerobic and anaerobic conditions. The same emission and excitation spectra were observed regardless of the bacterial cells grown aerobically or anaerobically when corresponding wavelengths of excitation monochromator and emission monochromator were set to 587 nm and 610 nm, respectively (Figure 2D). This demonstrates that the transition from anaerobic to aerobic environments does not affect the spectroscopic properties of mCherry fluorescent proteins.

### 3.2. R. palustris Mutants That Express RFP and Nitrogenase Simultaneously

**Figure 3:**
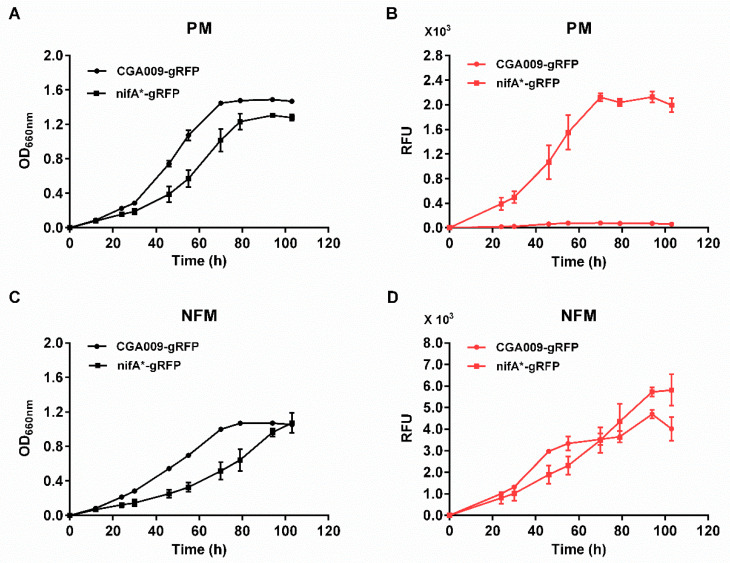
Measurement of growth and fluorescence of *R. palustris* strains expressing RFP. RFP was expressed in *R. palustris* CGA009 (CGA009-gRFP) and nifA* (nifA*-gRFP) strains grown in PM (**A**,**B**) and NFM (**C**,**D**), respectively. No fluorescence signals were detected in CGA009-gRFP grown in PM (**B**), a condition that does not express Mo nitrogenase. In contrast, nifA*-gRFP mutant expressing Mo nitrogenase constitutively under all growth conditions tested produced fluorescence when grown in both PM (**B**) and NFM (**D**). There was a positive correlation between fluorescence intensities and cell growths when *R. palustris* strains was able to express Mo nitrogenase and produce fluorescence. RFU, relative fluorescence unit. These data are the average of three independent experiments, and the error bars represent the standard deviation.

The nifA* mutant, a derivative of wild-type strain CGA009, has been developed by deleting 48 bp of the Q-linker region of the *nifA* gene to express Mo nitrogenase constitutively in both nitrogen-fixing medium (NFM) and photosynthetic medium (PM) [26,29]. The defined mineral medium NFM is the same as PM except for the lack of ammonium sulfate. To test if the fluorescence intensities are consistent with the expression levels of nitrogenase, we made a *mCherry*-inserted nifA* mutant strain (CGA3006), whose *mCherry* has the same context as *R. palustris* CGA3005. As shown in Figure 3, *R. palustris* CGA3005 (CGA009-gRFP), a strain that does not express nitrogenase in PM, only produced fluorescent signals in NFM but not in PM, while *R. palustris* CGA3006 (nifA*-gRFP), a strain that is able to derepress the expression of nitrogenase even in the presence of fixed nitrogen, produced fluorescence in both NFM and PM. The fluorescence intensities were positively correlated with cell growth. These results suggest that *R. palustris* strains CGA3005 and CGA3006 express RFP and nitrogenase simultaneously when they are incubated anaerobically with illumination.

### 3.3. RFP Can Be Used to Quantify Nitrogenase Gene Expression

**Figure 4:**
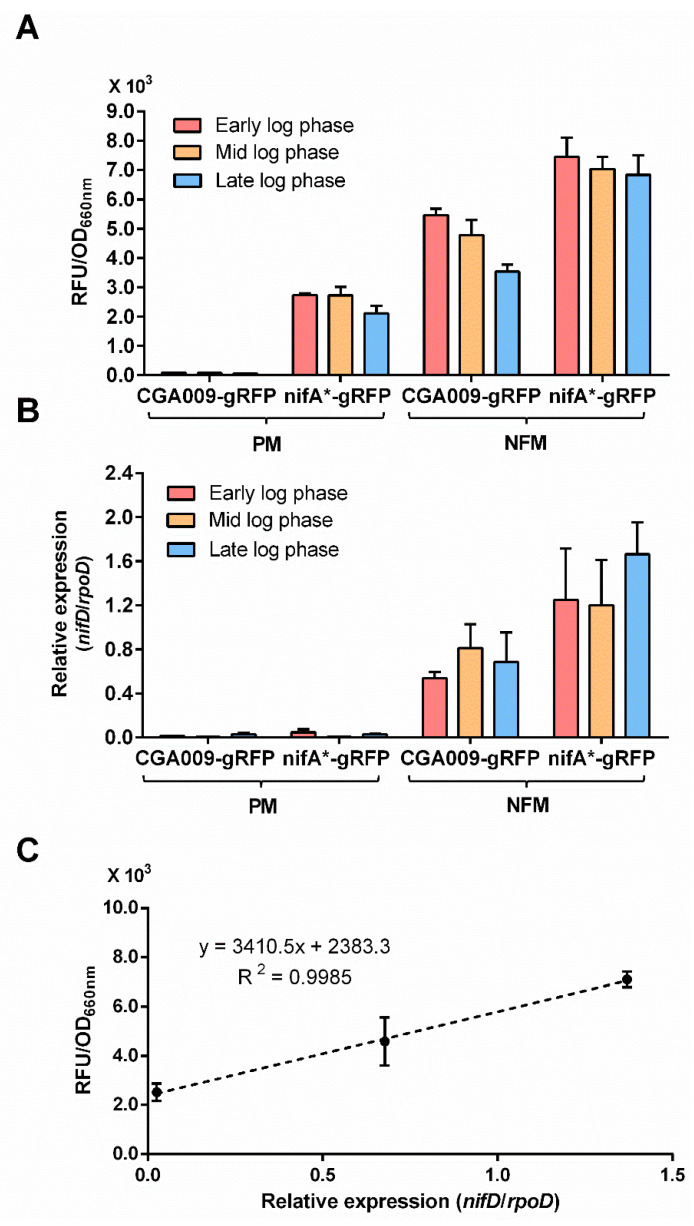
There is a strong correlation between relative fluorescence by RFP reporter system and relative expression by qRT-PCR in measuring nitrogenase gene expression. The relative fluorescence was quite stable during different stages of exponential growth in strains that express nitrogenase (**A**). We also measured the expression level of *nifD* gene encoding one of the three subunits of molybdenum nitrogenase by qRT-PCR. However, qRT-PCR method did not perform well on the measurement of low levels of nitrogenase expression (**B**). The nitrogenase gene expression measured by RFP reporter system was positively correlated with that determined by qRT-PCR (**C**). These data are the average of three independent experiments, and the error bars represent the standard deviation.

Ranging from early log to late log phases, fluorescence signals produced by *R. palustris* strains CGA3005 and CGA3006 did not show any significant fluctuations with the cell growth under conditions that express nitrogenase (Figure 4A), suggesting that nitrogenase gene expression is relatively stable if there is no environmental change. To further examine if the fluorescence intensities of RFP can reflect the nitrogenase gene expression accurately, we performed quantitative real-time PCR (qRT-PCR) to determine the nitrogenase gene (*nifD*) expression of *R. palustris* strains CGA3005 and CGA3006 at early, mid and late log phases. The same as the results obtained by RFP reporter system, no nitrogenase gene expression was detected by qRT-PCR in *R. palustris* CGA3005 grown with ammonium (Figure 4B). In addition, a good positive correlation was observed between the fluorescence intensity determined by RFP reporter system and the relative expression of the *nifD* gene obtained by qRT-PCR (Figure 4C). Though low levels of *nifD* expression could be detected in *R. palustris* CGA3006 grown with ammonium, an obvious fluctuation in *nifD* expression levels suggests RFP reporter system is superior to qRT-PCR in sensitivity (Figure 4B). The RFP reporter system is capable of measuring gene expression accurately even if the cell density is relatively low (Figure 4A), providing another advantage over qRT-PCR and β-galactosidase reporter system.

### 3.4. RFP Reporter System Can Be Used as an Efficient Tool for the Investigation of Transcriptional Regulation of Nitrogenase in Anaerobes

**Figure 5:**
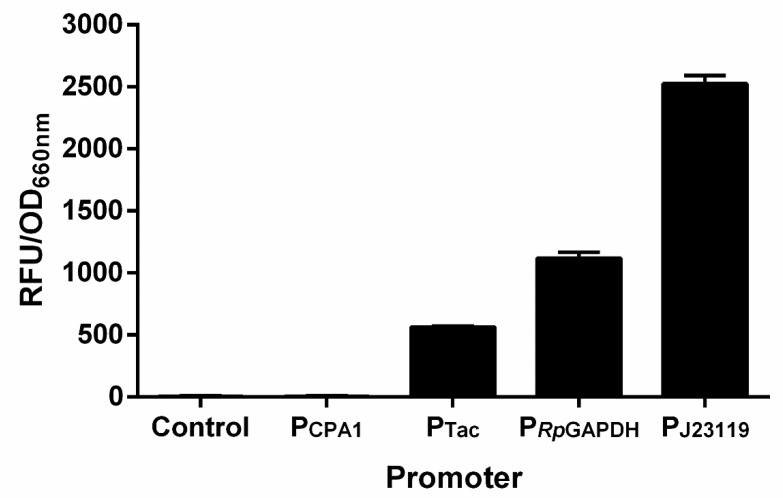
Measurement of promoter activity. A broad-host-range plasmid pBBR1MCS-5 was used to express *mCherry* gene under the control of P_CPA1_, P_Tac_, P_J23119,_ and P*_Rp_*_GAPDH_ promoters, respectively. After plasmid construction, the recombinant plasmids obtained were mobilized into *R. palustris* CGA009 by conjugation with *E. coli* S17-1. The promoter activities of P_CPA1_, P_Tac_, P*_Rp_*_GAPDH_, and P_J23119_ were determined by measuring the fluorescence intensities of *R. palustris* strains, finding out P_J23119_ had the highest activity among the promoters tested. These data are the average of three independent experiments, and the error bars represent the standard deviation.

**Figure 6:**
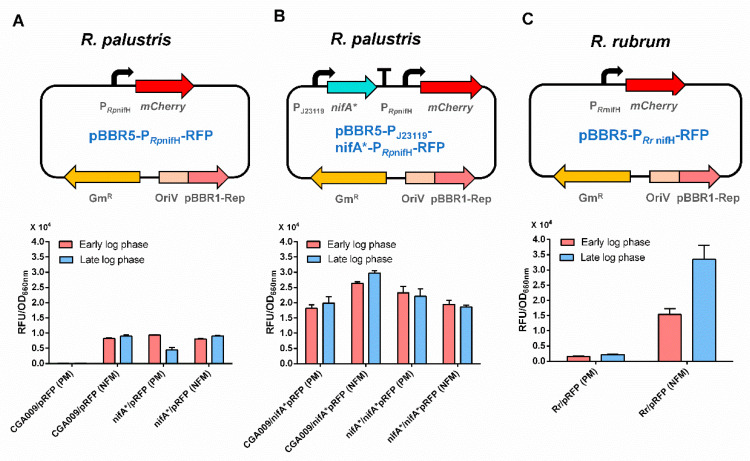
Plasmid-based RFP reporter system in photosynthetic bacteria. The promoter of *R. palustris nifH* gene (P*_Rp_*_nifH_), which requires an activator (active NifA or NifA*) to initiate transcription, was fused with *mCherry* gene to examine if an active NifA is present or not (**A**). When NifA* was overexpressed, *mCherry* gene will be expressed constitutively (**B**). In addition to *R. palustris*, this reporter system also can be used in another photosynthetic bacterium *R. rubrum* (**C**). The RFP reporter system developed here could be used to quantify gene expression in a wide range of photosynthetic bacteria grown anaerobically. These data are the average of three independent experiments, and the error bars represent the standard deviation.

It will be fast and convenient to study the nitrogenase regulation if a plasmid-based reporter system can be developed. Therefore, the *mCherry* gene was inserted into the broad-host-range plasmid pBBR1MCS-5 with P_CPA1_, P_Tac_, P_J23119_ and P_RpGAPDH_ promoters, respectively. The recombinant plasmids were mobilized into *R. palustris* CGA009 by conjugation with *E. coli* S17-1. Promoter P_J23119_ exhibited the strongest protomer activity among the four promoters tested, while P_CPA1_ was the weakest promoter (Figure 5). It provides us with more options for choosing promoters with appropriate activities. To examine if a plasmid-based RFP reporter system can be used to reflect the nitrogenase expression or not, we constructed a plasmid (pBBR5-P*_Rp_*_nifH_-RFP) expressing *mCherry* with the promoter of *R. palustris nifH* (P*_Rp_*_nifH_) (Figure 6A), which is activated by NifA under conditions without ammonium or by NifA* even in the presence of ammonium [26,29]. As expected, no fluorescence signals were detected in *R. palustris* CGA009 harboring pBBR5-P*_Rp_*_nifH_-RFP (CGA3007) grown in PM, a condition that makes NifA inactive due to the presence of ammonium. When an additional NifA* was overexpressed with a strong promoter P_J23119_ (CGA3009), high fluorescence intensities still could be detected in this CGA009-based strain grown with ammonium (Figure 6B). To determine if the RFP reporter system can be used in other anaerobic diazotrophs, we tested the RFP expression in *Rhodospirillum rubrum* grown in PM and NFM, respectively. Similar to the results obtained in CGA3007, fluorescence signals were only detected under conditions that express nitrogenase genes (Figure 6C). These results suggest that plasmid-based RFP reporter system genuinely reflects the nitrogenase gene expression, and can be used in a broad range of anaerobic bacteria.

## 4. Discussion

Diazotrophs have developed sophisticated mechanisms to control nitrogenase expression and activity, as the reduction of N_2_ to NH_3_ by nitrogenase is an energy-intensive process [34]. Photosynthetic bacterium *R. palustris*, which is widely distributed in the environment as a member of purple non-sulfur bacteria (PNSB), generates ATP required for nitrogenase activity from light by cyclic photophosphorylation under anaerobic conditions, making it attractive for the study of nitrogenase regulation [6,35]. Given that nitrogenase and many of the nitrogen regulators are oxygen sensitive, it is time-consuming and labor-intensive to study nitrogenase regulation *in vitro*. Therefore, in vivo assays play an important role in understanding the nitrogenase regulation of the photosynthetic diazotrophs [6]. However, the commonly used methods for in vivo study of nitrogenase expression such as qRT-PCR and *lacZ* reporter system are unable to measure nitrogenase gene expression in a timely manner [7,10]. Though Flavin-mononucleotide-based fluorescent proteins (FbFPs) work well for *E. coli* and *Bacteroides fragilis* as oxygen-independent fluorescence reporters [36,37], the background fluorescence of *R. palustris* at the emission wavelength of 492 nm (FbFPs) is 26-fold higher than that at 610 nm (mCherry). This means that mCherry will have a greater sensitivity than FbFPs if it is used as a fluorescence reporter in *R. palustris*. Therefore, we developed a simple and quick method for photosynthetic bacteria grown anaerobically to examine their nitrogenase gene expressions by RFP.

*R. palustris* only expresses nitrogenase under anaerobic conditions, but contradictorily, RFP needs molecular oxygen for its chromophore maturation. Oxygen access is of great importance for molecular oxygen to get into the fully folded barrel protein, but it may also trigger irreversible photobleaching and reduced photostability of a fluorescent protein [38]. In our experiments, the fluorescence signal of *R. palustris* CGA3005 can be fully recovered after a 4-h oxygen exposure, and is able to maintain the maximal fluorescence intensity for a couple of hours (Figure 2). It demonstrates that the immature RFP expressed under anaerobic conditions can fully mature and restore its fluorescence after it is exposed to air.

*R. palustris* develops a deep purple color as it synthesizes pigments for photosynthesis under anaerobic conditions [39]. When *R. palustris* were grown under conditions that repress the expression of nitrogenase, almost no fluorescence signals were detected (Figure 3 and Figure 4). That the background fluorescence was too low to be detected in the deep purple *R. palustris* demonstrates that the pigments *R. palustris* synthesized have no effect on the fluorescence detection. Moreover, the fluorescence of *R. palustris* can be easily measured in early growth stages at a low optical density at 660 nm (OD_660 nm_) of 0.2–0.3 (Figure 3), providing an advantage over the qRT-PCR and *lacZ* reporter systems. In addition, the expression level of nitrogenase in nifA* strains grown in PM was relatively low. In this situation, the qRT-PCR method was not sensitive enough to accurately measure the nitrogenase gene expression, but the RFP reporter system was still highly reliable (Figure 4). These results demonstrate that the RFP reporter system has an obvious advantage over qRT-PCR in sensitivity. Compared with the previously used *lacZ* reporter system in the photosynthetic bacterium *Rhodobacter capsulatus* [9,10], the RFP reporter system is operationally simple, time saving, and highly sensitive.

The plasmid-based RFP reporter system, which can be used in *R. palustris* without barriers (Figure 6), is quite useful for a quick test of potential nitrogenase regulators, and provides an important technical support for in vivo study of DNA-protein, protein-protein interactions that are similar to the yeast one-hybrid and yeast two-hybrid systems, respectively [40,41]. In addition to *R. palustris*, the RFP reporter system also worked well in another photosynthetic bacterium *R. rubrum* that has an Fe-only nitrogenase gene cluster [42], suggesting that this method can be widely used in photosynthetic bacteria.

## 5. Conclusions

Regulation of gene expression is of great significance for microorganisms to switch their lifestyle in response to the environmental changes. Compared with the commonly used qRT-PCR and *lacZ* reporter system, the RFP reporter system developed here is easy to operate and can quickly measure large amounts of samples with a high sensitivity, providing a better method for in vivo study of nitrogenase regulation in photosynthetic bacteria grown under anaerobic conditions. The RFP reporter system will contribute to a better understanding of the genetic regulation of nitrogenases, and can extend its applications to include other target genes and other anaerobic bacteria of interest.

## Figures and Tables

**Table 1:** Strains, plasmids, and primers used in this work.

Strain, Plasmid or Primer	Characteristics	Reference or Source
*E. coli* strains		
*E. coli* S17-1	*thi pro hdsR hdsM + recA*; chromosomal insertion of RP4-2 (Tc::Mu Km::Tn7)	[28]
*E. coli* P_J23119_-RFP	*E. coli* S17-1 harboring pBBR5-P_J23119_-RFP	This study
*R. palustris strains*		
CGA009	Wild type; *hupV* mutant; spontaneous frameshift (4-bp deletion) in *hupV*	[1]
CGA676	nifA*; 48-bp deletion encoding Q-linker amino acids 202–217; produces H_2_ in the presence of NH_4_^+^	[29]
CGA3005	CGA009-gRFP; CGA009 in which the RFP was introduced after its *nif* gene cluster using allelic exchange	This study
CGA3006	nifA*-gRFP; nifA* in which the RFP was introduced after its *nif* gene cluster using allelic exchange	This study
CGA3007	CGA009/pRFP; *R. palustris* CGA009 harboring pBBR5-P_nifH_-RFP	This study
CGA3008	nifA*/pRFP; *R. palustris* nifA* harboring pBBR5-P_nifH_-RFP	This study
CGA3009	CGA009/nifA*pRFP; *R. palustris* CGA009 harboring pBBR5-P_J23119_-nifA*-PnifH-RFP	This study
CGA3010	nifA*/nifA*pRFP; *R. palustris* nifA* harboring pBBR5-P_J23119_-nifA*-P_nifH_-RFP	This study
CGA3011	CGA009/P_J23119_-RFP; *R. palustri*s CGA009 harboring pBBR5-P_J23119_-RFP	This study
CGA3012	CGA009/P_CPA1_-RFP; *R. palustris* CGA009 harboring pBBR5-P_CPA1_-RFP	This study
CGA3013	CGA009/P_Tac_-RFP; *R. palustris* CGA009 harboring pBBR5-P_Tac_-RFP	This study
CGA3014	CGA009/P*_Rp_*_GAPDH_-RFP; *R. palustris* CGA009 harboring pBBR5-P*_Rp_*_GAPDH_-RFP	This study
*Rhodospirillum rubrum* UR2	Wild type; spontaneous Sm^R^ derivative of ATCC11170	[30]
*R. rubrum*/P*_Rr_*_nifH_-RFP	*R. rubrum* UR2 harboring pBBR5- P*_Rr_*_nifH_-RFP	This study
Plasmids		
pJQ200SK	Gm^R^, *sacB*; mobilizable suicide vector	[31]
pJQ-nif-RFP	Gm^R^, in-frame *nifHDK-mCherry* cloned into *Pst*I site of pJQ200SK	This study
pBBR1MCS5	Gm^R^, pBBR1 replicon, *mob^+^*; broad-host-range cloning vector	[27]
pBBR5-P_J23119_-RFP	Gm^R^, *mcherry* with J23119 promoter cloned into *Sac*I/*Eco*RI site of pBBR1MCS5	This study
pBBR5-P_CPA1_-RFP	Gm^R^, *mcherry* with CPA1 promoter cloned into *Sac*I/*Eco*RI site of pBBR1MCS5	This study
pBBR5-P_Tac_-RFP	Gm^R^, *mcherry* with Tac promoter cloned into *Sac*I/*Eco*RI site of pBBR1MCS5	This study
pBBR5-P*_Rp_*_Gapdh_-RFP	Gm^R^, *mcherry* with *R. palustris* CGA009 *GAPDH* promoter cloned into *Sac*I/*Eco*RI site of pBBR1MCS5	This study
pBBR5-P*_Rp_*_nifH_-RFP	Gm^R^, *mcherry* with *R. palustris* CGA009 *nifH* promoter cloned into *Sac*I/*Eco*RI site of pBBR1MCS5	This study
pBBR5-P_J23119_-nifA*-P*_Rp_*_nifH_-RFP	Gm^R^, P_J23119_-nifA* and P*_Rp_*_nifH_-RFP cloned into *Sac*I/*Eco*RI site of pBBR1MCS5	This study
pBBR5-P*_Rr_*_nifH_-RFP	Gm^R^, *mcherry* with *Rhodospirillum rubrum* UR2 *nifH* promoter cloned into *Sac*I/*Eco*RI site of pBBR1MCS5	This study
Primers		
nif-up-F	TTGATATCGAATTCCTGCAGGGCGTTCGTCGGCAGCC	
nif-up-R	CACCATATGTATATCTCCTTTCAGCGGATGATATCGAAGCTGACG	
nif-down-F	AGCTGTACAAGGCCGGCTAATGCCAAACGTTCGGACCAC	
nif-down-R	GTGGATCCCCCGGGCTGCAGTGTAGGCCTTGATCGCCGC	
Q-rpoD-F	CGTCCACTCGGTGCAGAAG	
Q-rpoD-R	GATGTTGCCTTCCTGAATGAG	
Q-nifD-F	AAGGTGATGCTGTATGTCGG	
Q-nifD-R	GCTGATAATCGTCGTTATGG	

## Data Availability

All datasets generated for this study are included in the article.
